# The influence of early research experience in medical school on the decision to intercalate and future career in clinical academia: a questionnaire study

**DOI:** 10.1186/s12909-017-1066-1

**Published:** 2017-12-11

**Authors:** Shona E. Boyle, Seonaidh C. Cotton, Phyo Kyaw Myint, Georgina Louise Hold

**Affiliations:** School of Medicine, Medical Sciences and Nutrition, University of Aberdeen, Foresterhill, Aberdeen, AB25 2ZD Scotland

**Keywords:** Intercalated degree, Early research exposure, Student research, Academic medicine, Clinical academic training

## Abstract

**Background:**

Currently, only one in three UK medical students undertake an intercalated degree. This has often been implicated as a result of financial obstacles or a lack of interest in research due to inadequate exposure to academic medicine. The aims of this study were to determine whether exposure to research early in medical school, through the initiation of an early years clinical academic training programme has a positive influence on the decision-making related to intercalating and a career long interest in research. This study also aims to evaluate the perceived views of the recipients of such a scholarship programme.

**Methods:**

All previous recipients of the Aberdeen Summer Research Scholarship (ASRS) (*n* = 117) since its inception in 2010 until 2015 were invited via email in June 2016, to take part in the survey. Data were analysed using SPSS for quantitative data and a thematic approach was used to derive themes from free text.

**Results:**

The overall response rate was 56% (66/117). Of the respondents, seven received the scholarship twice. Seventy-three percent were still at medical school and 26% were foundation doctors. One respondent indicated that they were currently not in training. Seventy percent of respondents have continued to be involved in research since completing the scholarship. Fifty percent embarked on an intercalated degree following the ASRS. Furthermore, two thirds of the respondents who were undecided about undertaking an intercalated degree before the scholarship, chose to intercalate after completing the programme. ASRS was generally thought of as a positive, influential programme, yet the success of individual ASRS projects was dependent on the allocated supervisors and the resources available for specific projects.

**Conclusions:**

Our findings indicate that early research exposure in medical school can provide students with a positive influence on involvement in research and allows students to make an informed decision about embarking on an intercalated degree. We therefore recommend the encouragement of similar programmes in medical schools to promote clinical academia at an early stage for medical students.

**Electronic supplementary material:**

The online version of this article (doi:10.1186/s12909-017-1066-1) contains supplementary material, which is available to authorized users.

## Background

Medical research encompasses an extensive variety of systematic investigation techniques. These range from basic research at a molecular level in the laboratory to clinical research that involves human participants. Clinical research covers a wide range of research methods from clinical epidemiology to clinical trials as well as clinical translational research which may involve medical technologies and related disciplines such as medical physics; it primarily concerns with better understanding of health and disease through identification of risk, determination of prognosis, assessment of management options and developing treatments for medical conditions. Medical research is an integral part of advancing medicine and a prerequisite for a clinical academic career path. However, medicine is predominantly an undergraduate degree in the UK and as a result, students will have often had minimal research experience before commencing medical school.

Indeed, intercalated degrees and summer research projects, out with term time, are frequently the only research exposure opportunities available to medical students [1]. An intercalated degree is an academic opportunity in medical school; it is a taught programme that allows a student to take a year out of their medical degree to study a specific area of interest. Students may have the opportunity to study in an area of basic science, education and teaching or clinical research (to achieve a BSc or postgraduate MSc or MRes).

Early research exposure, clinical or otherwise, is highly valued in a clinical academic career; medical students have reported that gaining early research experience provides an advantage when applying for specialty training and helps them to perform better in interviews and gives evidence of an interest in a specific speciality [2]. Furthermore, it has long been reported that students who choose to intercalate are more interested in medical research than their peers [3]. Short-term benefits of intercalating include improved exam results and academic performance in the years following their intercalated degree [4].

Taking into consideration the benefits mentioned, questions as to why only one third of medical students in the UK pursue an intercalated degree and why these numbers are declining have been highlighted [3, 5]. Students have most commonly reported that their decision not to intercalate was due to financial obstacles or a lack of interest in research due to inadequate exposure to medical research [6, 7]. While the most frequent reasons for not intercalating, financial obstacles, are somewhat out with the university control, a lack of exposure to research is something that can be addressed throughout universities in the UK.

In Aberdeen, there is a competitive intercalating programme which allows 3rd and 4th year medical students to pursue a BSc, MSc or MRes with a taught and research component. However, before the decision to intercalate arises, the university presents students with the opportunity to gain experience in a research environment at the end of their 1st and 2nd year. A similar programme is available in Dundee: DCAT vacation studentships, predominantly advertised to 2nd and 3rd years. Other comparative programmes exist at UK universities such as UCL and the London School of Medicine and Dentistry but these are open scholarships to any UK medical student, while Aberdeen and Dundee offer internal scholarships.

The Aberdeen Summer Research Scholarship (ASRS) Programme at the University of Aberdeen was launched in 2010 with the intention of creating clinical research opportunities for 1st and 2nd year medical students to facilitate development and understanding of clinical research. It was also intended to build a life-long engagement of research within their preferred clinical subject area. This is an 8-week programme that provides students with the opportunity to understand more about the work of clinical academics – seeing both sides of their role, namely clinical duties and academic research.

The programme is a competitive scheme advertised to all 1st and 2nd year medical students. Prior research experience is not a requirement. Selection is through an interview process by a panel of two senior academics and successful students are allocated to an academic supervisor who normally provides a scholarship stipend and project, which will be carried out in an 8-week period.

ASRS was developed essentially as a mentoring programme to provide research skills training in either wet or dry projects as well as the opportunity to undertake clinical observations early in their training. Wet projects in the laboratory allow an inexperienced participant to be familiarised with basic molecular biology skills such as pipetting, polymerase chain reaction (PCR) and Western Blot while those with some degree of experience, have a chance to translate their experimental results to clinical settings. Whereas, dry projects focus on more desk-based skills such as systematic reviews, data driven projects with relevant statistical training in an epidemiological or clinical project. Furthermore, all projects present the opportunity to develop general skills such as project design and execution, leadership, and management skills. At the end of the 8 weeks, all ASRS participants are expected to have achieved learning objectives set out jointly with the assigned supervisors at a level appropriate to their academic potential; thus they are individualised and it is a unique aspect of ASRS programme. No other outcomes are mandatory for this programme except either poster or oral presentation in November of the same year in the ASRS evening symposium, however, many students have chosen to continue working on their project to try and achieve publications and presentations at a national and international level.

Despite the scholarship programme having been in existence for 6 years, it had not formally been evaluated. To our knowledge, a formal evaluation of any similar medical school research programme has not been published. Prior to the commencement of this programme, the University of Aberdeen reported that between 2004 and 2007, only 17.9% of medical students chose to carry out an intercalated degree each year and in 2007/8 this number was as low as 12.9% [5].

In view of this, we set out to formally survey the views of Aberdeen University medical students who entered into the ASRS programme and whether the programme encouraged further involvement in research, specifically the decision to intercalate. We also evaluated the achievements they attained during and after the programme, including publications and presentations of work at a national and international level. We also specifically explored benefits and disadvantages of the format of the programme.

## Methods

To achieve aforementioned aims we conducted a questionnaire survey to previous recipients of the ASRS programme (Additional file 1). The survey was commissioned by the Aberdeen Clinical Academic Training Executive Board and NHS North of Scotland Ethics committee satisfied that the project did not require ethical approval.

### Participants

The ASRS programme database contains contact information in the form of student email addresses of all ASRS recipients. All recipients who had completed the programme at the time of survey distribution were invited to take part (i.e. recipients completing the programme during 2010–2015).

### Data collection

Survey questions covered four main themes; demographic information, student experience of their individual ASRS project, output generated as a result of the programme, student’s overall satisfaction with the ASRS programme and recommendations for the future. Students may have completed two projects (one in their first year and one in their second year), and the survey was designed to be able to capture information for two ASRS projects.

The survey was semi-qualitative and contained closed and open questions. The survey was piloted before use. It was administered electronically using the questionnaire software SNAP. This software was used to generate and anonymise the survey and its responses. The invitation to take part in the survey was sent in June 2016 via email; the email contained an internet web link to the questionnaire. Participants were given a 3-week period to return completed questionnaires from the date of receipt. Two email reminders were sent: one ten days after the date of the initial invitation and a second, 1 week after that (1 week before the survey closed). A copy of the questionnaire is available on request from the authors.

### Data handling and analysis

Data were directly exported from SNAP to Microsoft Excel 2013. Quantitative data were analysed using SPSS software (IBM SPSS Statistics 24) while qualitative data of open questions/responses were coded using a thematic approach. Common themes were identified for each open question. These themes were then charted and interpreted.

## Results

In the 6 years since the ASRS programme had been initiated, 117 students had participated in the scheme, 56 male and 61 female (Table 1). The lowest number of scholarships were awarded in the initial 2 years, whilst the programme was being set up.

**Table 1:** Response rates, by demographic characteristics

		ASRS recipients (n)	Survey respondents (n)	Response Rate (%)
Gender	Male	56	41	73.2%
	Female	61	25	41.0%
Year when ASRS undertaken	2010	14	3	21.4%
	2011	15	12	80.0%
	2012	26	20	76.9%
	2013	20	15	75.0%
	2014	20	9	45.0%
	2015	22	15	68.2%
Overall		117	66	56.4%

Following one initial email and two reminder emails over the course of 3 weeks, the overall response rate was 56%. Response rates were higher among male students and lowest amongst those who had completed their first ASRS in 2010 (Table 1).

62% of respondents were male and 38% female (Table 2). Before commencing the programme, the majority of respondents (80%) had high school qualifications only; the remainder also had a university degree (17% undergraduate degree, 3% MSc). The median age of respondents at the start of the programme was 20.

**Table 2:** Respondents demographic summary

Demographics		Respondents *n* = 66
Gender	Male	41 (62%)
	Female	25 (38%)
Qualification prior to ASRS	High School	52 (80%)
	Undergraduate degree	11 (17%)
	MSc	2 (3%)
	PhD	0
	Missing^a^	1
Current stage of training	Medical Student	48 (73%)
	Foundation Doctor	17 (26%)
	Core Trainee	0
	Speciality Trainee	0
	Other	1 (1%)
Age when started ASRS	18	3 (4.5%)
	19	18 (27%)
	20	20 (30%)
	21	6 (9%)
	22	3 (4.5%)
	23	6 (9%)
	24	4 (6%)
	25	1 (1.5%)
	26	2 (3%)
	27	1 (1.5%)
	36	1 (1.5%)
Year of study^b^	2010	3 (4%)
	2011	12 (16%)
	2012	20 (27%)
	2013	15 (20.5%)
	2014	9 (12%)
	2015	15 (20.5%)

^a^Missing data excluded from denominator when calculating %

^b^ *N* = 73

Seven students reported that they had completed two ASRS projects – one in first year and one in second year.

**Fig. 1:**
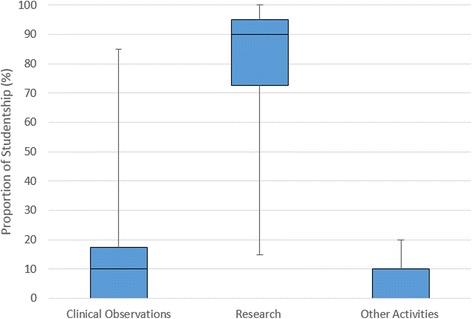
Proportion of time spent between clinical observations, research and other activities during ASRS scholarship. Other activities include administration work, attending meetings etc. Boxes represent the middle 50% of responses with the median represented by the middle line and the tails represent the maximum and minimum values collected

Respondents were asked to specify the proportion of time they spent doing the following tasks: research; clinical observation; other, during their individual project. On average, there was a balance of 90% research, 10% clinical observations during individual projects (Fig. 1). Eighty percent of individual projects involved clinical observations in their associated research area. This balance of research and clinical exposure was largely well received by students. One respondent summarised their feelings on the research balance as:

‘I enjoyed the amount [*clinical exposure*] that was required to put everything into context but was glad that the main focus was research’.

Although some respondents (20%) felt they could have benefitted from more clinical exposure related to their project, 73% of respondents were happy with the research-dominated balance with comments including:

‘It was made clear at the start that the aim of the project was to gain research experience’.

**Fig. 2:**
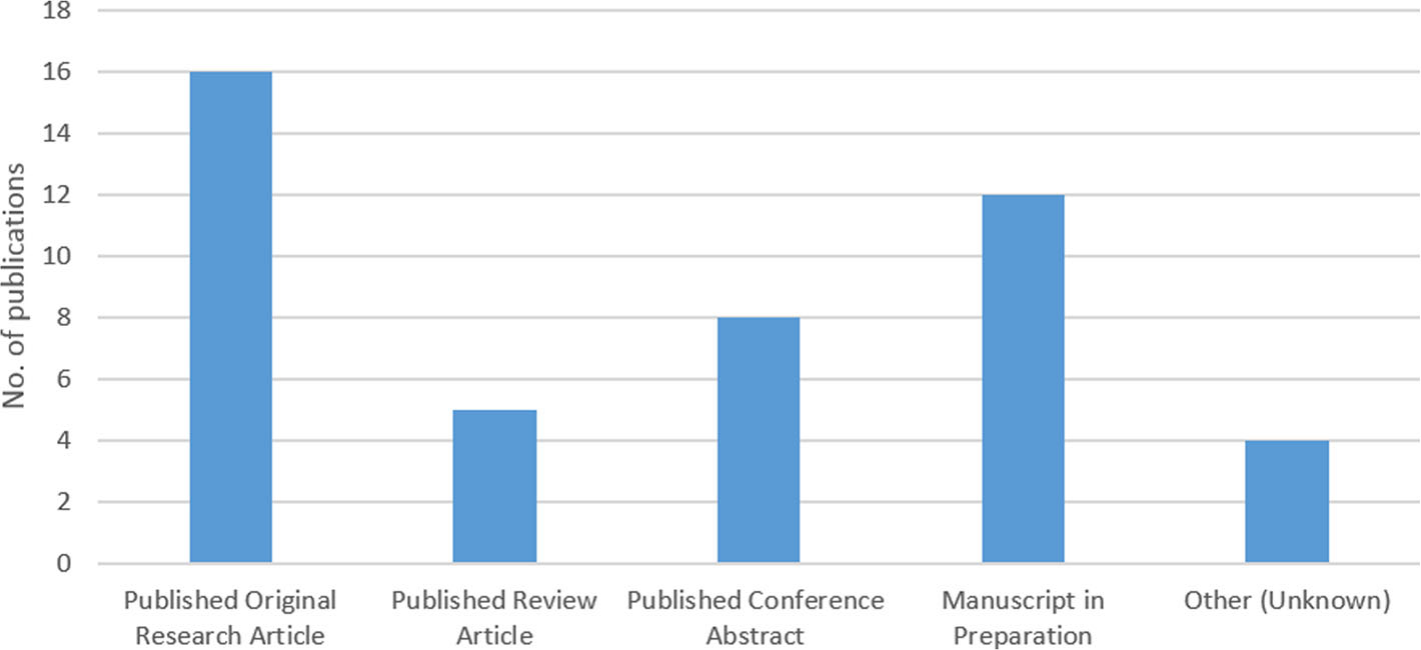
Number of written publications produced as a result of ASRS projects. Self-reported data from the survey

In total, students reported that their ASRS project work had contributed to 29 presentations at national meetings/conferences; nine presentations at international meetings/conferences; 16 original research papers and five review papers (Fig. 2). In addition, they reported 12 manuscripts in preparation.

Some projects had more than one output, and considering the highest level of output from the project; 21 projects resulted in an original or review paper; five in a presentation at an international meeting/conference; eight in a presentation at a national meeting/conference; two in a conference abstract; while 37 of the projects did not have any such outputs.

**Fig. 3:**
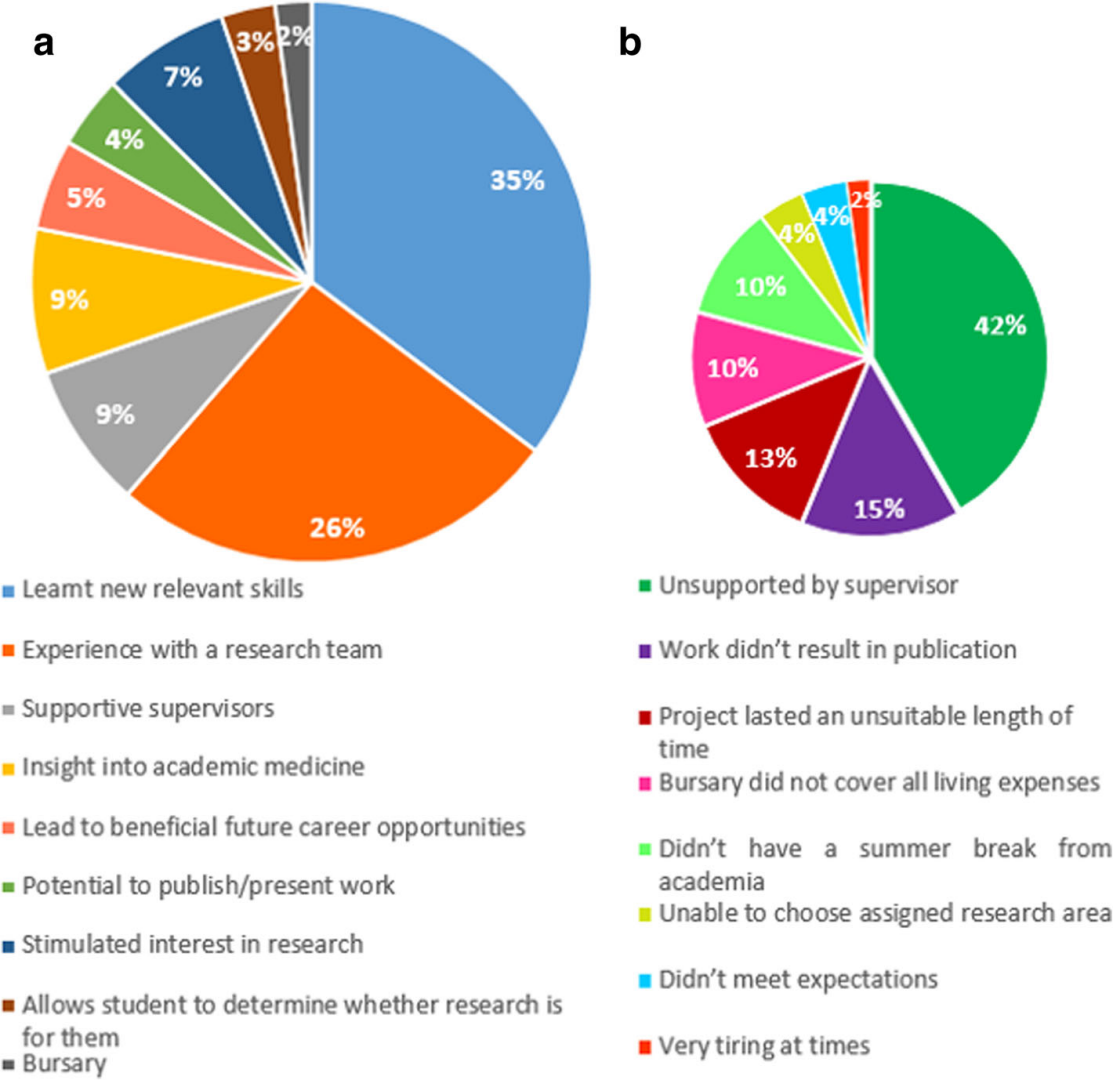
Survey recipient reported benefits and drawbacks of ASRS early research programme. **a** Benefits reported, *N* = 96. **b** Disadvantages reported, *N* = 48

Respondents were asked to comment on the overall benefits and drawbacks of the programme through open questions. Of the 96 reported benefits of the programme, 34 (35%) were associated with learning new skills (data handling and analysis, lab techniques etc.), with a further 25 relating to the benefits of gaining experience within a research team (26%) (Fig. 3a).

Twenty-two of the respondents (34%) stated that there were no drawbacks to their ASRS involvement. However, 20 of the 48 (42%) disadvantages reported were associated with feeling they could have been more supported by their allocated supervisor for varying reasons (Fig. 3b).

40% (14/35) of the improvements suggested by respondents were supervisor dependent (Table 3). Comments covered positive supervisor experiences e.g.:

‘Perhaps I struck gold with my placement but I feel that there was nothing lacking. I feel that the key ingredient to a successful ASRS would be involving excellent supervisors’

While others identified negative supervisor experiences, e.g.:

‘Didn't feel supported enough, in over my head, poor relationship between myself and mentor’.

The most commonly suggested improvements included more a formal structure to the programme, for example an introductory session or group tutorials before commencement of projects, making sure supervisors have clear instructions as to their role, and group tutorials (Table 3).

**Table 3:** Survey recipients’ suggestions for programme improvements

Suggested Improvements	
Lack of Resources	Group tutorials on research techniques would have been useful
	It would be good to be able to choose what research area to work in
	Make ASRS available to more students
	Provide more incentives, such as local presentations
Supervisor Dependent Issues	Provide more insight into the publication process
	More clinical exposure
	Provide more achievable projects within the time frame
	Students should have the opportunity to continue working on projects after the 8 weeks
	Projects should involve more current research
	Provide allocated desk space
Overall Programme Improvements	Have a clearer programme structure for students and supervisors
	Should have an introductory meeting with other ASRS recipients and staff
	Feedback session straight after ASRS from students

**Fig. 4:**
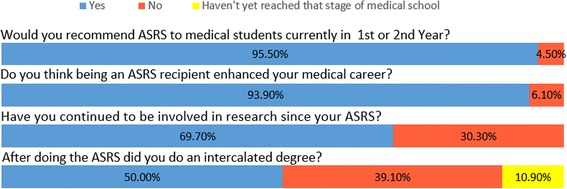
Student views of ASRS programme following project completion. First two questions: *N* = 66, Last two questions: *N* = 64

More than 95% (63/66) of respondents would recommend ASRS to medical students currently in 1st and 2nd year and 94% (62/66) felt it had enhanced their medical career (Fig. 4). Furthermore, 70% (44/64) of respondents have continued to be involved in research since ASRS, some continuing research with the same department as their ASRS mentor, others have carried out further research projects in other university or clinical areas.

### Early research opportunities positively influence student decision to intercalate

**Fig. 5:**
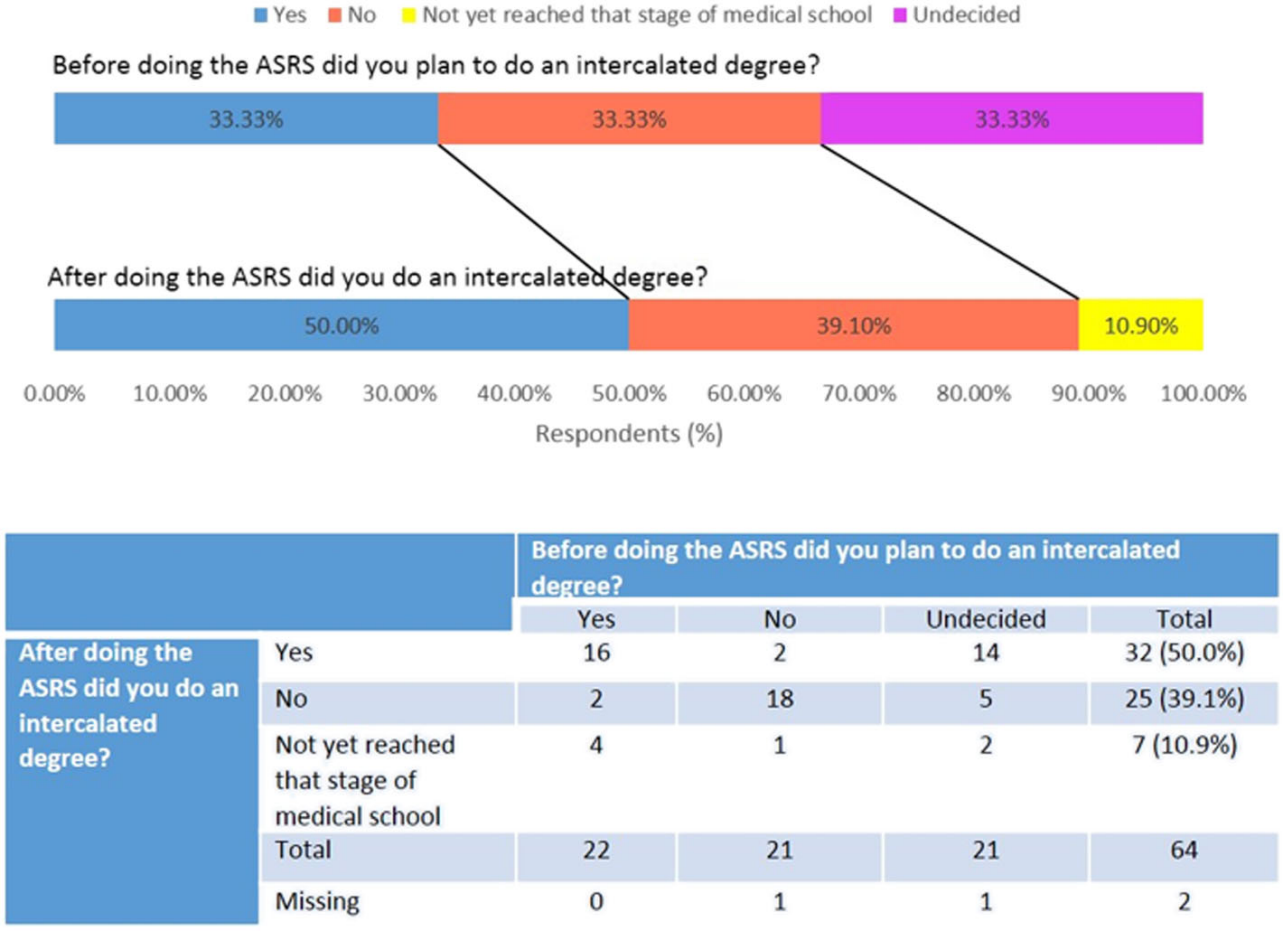
Student views on doing an intercalated degree before and after ASRS. N = 64

50% (32/64) of respondents chose to undertake an intercalated degree following their ASRS experience (Fig. 4). Furthermore, of the respondents who had not decided whether they would undertake an intercalated degree before ASRS, 67% (14/22) of them chose to intercalate following the research experience during this programme (Fig. 5). Moreover, two respondents that did not intend to intercalate before ASRS, changed their mind after their ASRS experience and chose to undertake an intercalated degree.

It is important to note that two respondents who intended to undertake an intercalated degree before ASRS decided not to following the programme. There are some common features in the questionnaire responses of these two respondents. Both their projects involved 100% research with no clinical exposure. Moreover, their projects did not result in publication and they both felt unsupported by their supervisors. However, both recipients have continued to be involved in research in other ways and would still recommend the programme to their peers and felt that the experience enhanced their medical school career. When asked why they decided not to intercalate, one of the two recipients stated that ‘Projects on offer were not of interest’ and the other did not comment. In contrast, two respondents who did not intend to intercalate before ASRS changed their mind after their ASRS experience and chose to undertake an intercalated degree. One of these respondents stated that their reason for intercalating was due to a ‘positive ASRS research experience’. Both of these respondents felt adequately supported by their supervisor and published an original research article as a result of their ASRS projects.

Respondents were also asked to explain why they chose to undertake an intercalated degree. The most commonly reported reason for choosing to intercalate was an interest in developing research experience in general or a specific field as a result of the ASRS programme (Table 4). Reasons for not intercalating included financial barriers or respondents already holding a BSc degree (Table 4).

**Table 4:** Survey recipient’s reasons for intercalating and not intercalating

Reasons for intercalating	Responses (*n* = 35^a^)
To develop research skills after positive ASRS experience	11
To gain an extra degree	4
To pursue interest in a specific field	4
To determine whether a future in research is right for them	2
To have a break from medicine	1
To pursue interest in academic medicine	2
To pursue interest in medical education	1
No reason given	10
Reasons not to intercalate	Responses (*n* = 26^b^)
Financial reasons	11
Already had a BSc	5
Personal reasons	3
Focused on clinical skills	2
No interest in topics offered	2
Too long at university	1
No reason given	2

^a^ *N* > 32 some respondents gave more than one reason for intercalating

^b^ *N* > 25 because some respondents gave more than one reason for not intercalating

## Discussion

This survey has established that Aberdeen University’s early research programme provides a positive influence in recipients’ decision-making to engage in research and importantly to intercalate, with 50% of recipients going on to pursue an intercalated degree, which is higher than the average proportion at the University of Aberdeen (17.9%) [5]. Furthermore, of the recipients that were unsure about intercalating, two thirds of them made the decision to intercalate following ASRS. This suggests that an early exposure to research in medical school allows students to make an informed decision on undertaking an intercalated degree, which in the future may lead to a career in academic medicine. It could be argued that those who do programmes such as ASRS, are more likely to intercalate as they are demonstrating an interest in research early on in medical school, however this experience gives individuals an opportunity to experience research in advance of making a decision about intercalating.

A study in Auckland found that only 8.6% of medical students displayed an interest in intercalating. The main reasons for such low interest rates included a lack of interest as well as social and financial reasons [6]. Focusing on the former, a lack of interest may stem from an absence of research exposure before students are presented with the opportunity to intercalate [7]. This highlights the importance of early research opportunities in medical school to help develop an interest in academic research.

Reasons not to intercalate have been previously reported from a questionnaire based study involving University of Aberdeen medical students in 2010, before the ASRS programme commenced. The published study found that the financial burden and a lack of interest in an extra year of study were the two most common reasons for not intercalating [5]. Our study shows that financial barriers remain the most commonly reported reason for not intercalating, particularly for international students within this cohort of medical students.

It has been found that the beneficial effects of an intercalated degree are diminished in medical schools where the majority of students intercalate, possibly explained by resource dilution [3]. The authors of that study postulated that appropriate staff and other resourcing of intercalated degrees may be integral to their success. Additionally, it has been suggested that clinical academics are desirable mentors for intercalated degrees with their students achieving a greater number of first class honours awards and presenting more posters and publications from their projects [8]. From the suggestions collected in our survey, it would appear that resources and enthusiasm of supervisors are similarly important for the success of an early research programme like ASRS. Chang & Ramnanan [9] reviewed 20 self-reported medical student experiences in university research programmes and found that students felt research experience stimulated an interest in medical research and provided them with relevant research skills. They concluded that programmes like this could be improved with similar ideas stated by our survey, such as: effective student-mentor relationship, acknowledgement of student contribution and the option to extend the duration of the research experience [9].

Furthermore, research activity during medical school benefits both the student and the institution as it aids the student in their future career, while their institutions benefit from an increase in their research output through student publications [10].

### Possible limitations

There are some potential limitations of this study. The response rate was 56%. Response was lowest from those in the first year of the ASRS cohort – this may be explained by an increased difficulty in correspondence, as they will no longer be students or regularly using their Aberdeen University email address. The majority of responses were from males which does not reflect the demographic of ASRS recipients. Some survey recipients were still students at the time they completed the survey. However, the survey was anonymous, and we believe this allowed respondents to give open and honest answers.

## Conclusions

As well as its positive influence on intercalation, this survey has demonstrated that early university research programmes such as ASRS are overall a very positive experience for students. It gives students that may have no previous research experience the opportunity to develop an appreciation of clinical research and may help them to determine early on in their medical school careers whether research is something they wish to engage with. Ultimately, it also allows students to make an informed decision when it comes to the time in their university career when they must choose whether to intercalate or not. We recommend the encouragement of similar programmes in medical schools to promote clinical academia at an early stage for medical students.

## Additional file

Additional file 1: Aberdeen Summer Research Studentship Programme Survey. ASRS Survey. Survey form distributed to participants. (PDF 115 kb)

## Abbreviations

ASRS: Aberdeen Summer Research Scholarship

## References

[1] Funston GM, Young AM (2012). Action is required to safeguard the future of academic medicine in the UK. Nat Med.

[2] Mabvuure NT (2012). Twelve tips for introducing students to research and publishing: a medical student's perspective. Med Teach.

[3] McManus IC, Richards P, Winder BC (1999). Intercalated degrees, learning styles, and career preferences: prospective longitudinal study of UK medical students. BMJ.

[4] Cleland JA, Milne A, Sinclair H, Lee AJ. An intercalated BSc degree is associated with higher marks in subsequent medical school examinations. BMC Med. Educ. 2009;9(24) 10.1186/1472-6920-9-24 PMC2689211 19454007

[5] Nicholson JA, Cleland J, Lemon J, Galley HF (2010). Why medical students choose not to carry out an intercalated BSc: a questionnaire study. BMC Med Educ.

[6] Park SJ, Liang MM, Sherwin TT, McGhee CN (2010). Completing an intercalated research degree during medical undergraduate training: barriers, benefits and postgraduate career profiles. NZ Med J.

[7] Rushforth B (2004). Academic medicine and intercalated degrees-the myth of student choice. Med Educ.

[8] Stubbs TA, Lightman EG, Mathieson P. Is it intelligent to intercalate? A two centre cross-sectional study exploring the value of intercalated degrees, and the possible effects of the recent tuition fee rise in England. BMJ Open. 2013;3:e002193 10.1136/bmjopen-2012-002193 PMC3563132 23355672

[9] Chang Y, Ramnanan CJA (2015). Review of literature on medical students and scholarly research: experiences, attitudes, and outcomes. Acad Med.

[10] Jacobs CD, Cross PC (1995). The value of medical student research: the experience at Stanford University School of Medicine. Med Educ.

