# Supplementary material for: The influence of early research experience in medical school on the decision to intercalate and future career in clinical academia: a questionnaire study
Source: BMC Med Educ. 2017 Dec 11;17:245. doi: 10.1186/s12909-017-1066-1 (PMC5725945; doi:10.1186/s12909-017-1066-1)
Supplement: Additional file 1: — Aberdeen Summer Research Studentship Programme Survey. ASRS Survey. Survey form distributed to participants. (PDF 115 kb) [file 12909_2017_1066_MOESM1_ESM.pdf]

Aberdeen Summer Research Studentship Programme Survey

PART A - DEMOGRAPHICS

Gender

- ❑ Male
- ❑ Female
- ❑ Prefer not to say

What qualifications did you have prior to undertaking the ASRS? (Tick the highest level)

- ❑ High school or equivalent
- ❑ Undergraduate degree (BSc or equivalent)
- ❑ MSc
- ❑ Phd

Current stage of training

- ❑ Medical student
- ❑ Foundation doctor
- ❑ Core trainee
- ❑ Speciality trainee
- ❑ Other

Other than MBChB, have you gained or started any other academic qualifications after ASRS?

- ❑ No
- ❑ Yes

If yes, please state the qualification and year(s) of study. ______________________________

When did you apply for the ASRS?

- ❑ 1st year only
- ❑ 2nd year only
- ❑ Both years

When were you awarded an ASRS place?

- ❑ 1st year only
- ❑ 2nd year only
- ❑ Both years

During which year(s) did you undertake the ASRS? (Tick all that apply, and state the age you were when you did your first ASRS)

- ❑ 2010
- ❑ 2011
- ❑ 2012
- ❑ 2013
- ❑ 2014
- ❑ 2015

Age at the start of first ASRS

❑ 17	❑ 26	❑ 35	❑ 44
❑ 18	❑ 27	❑ 36	❑ 45
❑ 19	❑ 28	❑ 37	❑ 46
❑ 20	❑ 29	❑ 38	❑ 47
❑ 21	❑ 30	❑ 39	❑ 48
❑ 22	❑ 31	❑ 40	❑ 49
❑ 23	❑ 32	❑ 41	❑ 50+
❑ 24	❑ 33	❑ 42	
❑ 25	❑ 34	❑ 43	

PART B - PROCESS & OUTPUTS (FIRST ASRS CYCLE)

If you have completed ASRS in both years, please complete the following section with respect to your **first ASRS programme only**

Did you have a named academic supervisor for your ASRS?

- ❑ No
- ❑ Yes
- ❑ Unsure

Did you feel adequately supported by your academic supervisor and their team?

- ❑ No
- ❑ Yes

When were the expected outcomes of the ASRS made clear?

- ❑ At the the beginning of the ASRS
- ❑ During the ASRS
- ❑ Never

Whilst doing the ASRS, what proportion of your studentship **(should total 100%)** was spent doing:

Clinical Observations (%) ________________

Research (%) ________________

Other activities (%) ________________

Do you feel this balance was appropriate?

- ❑ No
- ❑ Yes

Please explain your answer ____

What clinical specialty were you attached to?

____

What type of research project were you involved in?

- ❑ Lab
- ❑ Non-Lab

What did your project involve? (Describe in 50-100 words)

Did you continue working on the project beyond the funded ASRS period?

- ❑ No
- ❑ Yes

Have you presented work you undertook during the ASRS at a meeting or conference?

- ❑ No
- ❑ Yes - Local meeting or conference (e.g. within hospital)
- ❑ Yes - National meeting or conference (Scotland/UK)
- ❑ Yes - International meeting or conference

If yes, please state how many presentations and what type (e.g. 2 oral, 1 poster)

Have other members of the research team presented the work you undertook during the ASRS at a meeting or conference?

- ❑ No
- ❑ Yes - Local meeting or conference (e.g. within hospital)
- ❑ Yes - National meeting or conference (Scotland/UK)
- ❑ Yes - International meeting or conference

If yes, please state how many presentations and what type (e.g. 2 oral, 1 poster)

Has the work you undertook during the ASRS led to a successful publication?

- ❑ No
- ❑ Conference abstract
- ❑ Review article
- ❑ Original research article
- ❑ Other

If yes, please state how many publications, what type and your role (e.g. first author, co-author, acknowledged as contributor)

Are there any manuscripts in preparation or under review that include the work you undertook during the ASRS?

- ❑ No
- ❑ Yes

If yes, please state how many publications, what type and your role (e.g. first author, co-author, acknowledged as contributor)

To what extent did this ASRS impact on your subsequent learning?

- ❑ Major negative impact
- ❑ Minor negative impact
- ❑ No impact
- ❑ Minor positive impact
- ❑ Major positive impact

Please explain your answer ______________________________

	Very Dissatisfied	Dissatisfied	Neither dissatisfied or satisfied	Satisfied	Very satisfied
Please rate how satisfied you are with the overall experience of this ASRS	❑	❑	❑	❑	❑

PART B2 - PROCESS & OUTPUTS (SECOND ASRS CYCLE)

N.B. If you have not yet completed your second ASRS, please leave this section blank.

Please complete Part B2 with respect to your ASRS programme **in second year only**

Did you have a named academic supervisor for your ASRS?

- ❑ No
- ❑ Yes
- ❑ Unsure

Did you feel adequately supported by your academic supervisor and their team?

- ❑ No
- ❑ Yes

When were the expected outcomes of the ASRS made clear?

- ❑ At the the beginning of the ASRS
- ❑ During the ASRS
- ❑ Never

Whilst doing the ASRS, what proportion of your studentship **(should total 100%)** was spent doing:

Clinical Observations (%) ____________

Research (%) ____________

Other activities (%) ____________

Do you feel this balance was appropriate?

- ❑ No
- ❑ Yes

Please explain your answer ______________________________

What clinical specialty were you attached to?

What type of research project were you involved in?

- ❑ Lab
- ❑ Non-Lab

What did your project involve? (Describe in 50-100 words)

Did you continue working on the project beyond the funded ASRS period?

- ❑ No
- ❑ Yes

Have you presented work you undertook during the ASRS at a meeting or conference?

- ❑ No
- ❑ Yes - Local meeting or conference (e.g. within hospital)
- ❑ Yes - National meeting or conference (Scotland/UK)
- ❑ Yes - International meeting or conference

If yes, please state how many presentations and what type (e.g. 2 oral, 1 poster)

Have other members of the research team presented the work you undertook during the ASRS at a meeting or conference?

- ❑ No
- ❑ Yes - Local meeting or conference (e.g. within hospital)
- ❑ Yes - National meeting or conference (Scotland/UK)
- ❑ Yes - International meeting or conference

If yes, please state how many presentations and what type (e.g. 2 oral, 1 poster)

Has the work you undertook during the ASRS led to a successful publication?

- ❑ No
- ❑ Conference abstract
- ❑ Review article
- ❑ Original research article
- ❑ Other

If yes, please state how many publications, what type and your role (e.g. first author, co-author, acknowledged as contributor) ______________________

Are there any manuscripts in preparation or under review that include the work you undertook during the ASRS?

- ❑ No
- ❑ Yes

If yes, please state how many publications, what type and your role (e.g. first author, co-author, acknowledged as contributor) ______________________

To what extent did this ASRS impact on your subsequent learning?

- ❑ Major negative impact
- ❑ Minor negative impact
- ❑ No impact
- ❑ Minor positive impact
- ❑ Major positive impact

Please explain your answer ______________________

	Very Dissatisfied	Dissatisfied	Neither dissatisfied or satisfied	Satisfied	Very satisfied
Please rate how satisfied you are with the overall experience of this ASRS	❑	❑	❑	❑	❑

PART C - AFTER THE ASRS CYCLE

Before doing the ASRS did you plan to do an intercalated degree?

- ❑ No
- ❑ Yes
- ❑ Hadn't decided

After doing the ASRS did you do an intercalated degree?

- ❑ No
- ❑ Yes
- ❑ Not yet reached that stage of medical school

Why did you decide to do an intercalated degree? ______________________

Why did you decide not to do an intercalated degree?

Are you considering applying to do an intercalated degree?

- ❑ No
- ❑ Yes

Please explain your answer

Did you do your intercalated degree within the same research speciality as your ASRS?

- ❑ No
- ❑ Yes

Have you continued to be involved in research since your ASRS?

- ❑ No
- ❑ Yes

If yes, what type of research have you been involved in? (Please describe in up to 100 words)

PART D - EVALUATION OF THE ASRS EXPERIENCE

Please describe any benefits of your ASRS experience. If none - please state "none".

Please describe any drawbacks of your ASRS experience. If none - please state “none”.

Do you think being an ASRS recipient enhanced your medical school experience?

- ❑ No
- ❑ Yes

Please explain your answer ____________________

Would you recommend ASRS to medical students currently in 1st or 2nd year?

- ❑ No
- ❑ Yes

Please explain your answer ____________________

Please provide any other feedback which may be useful to further develop the ASRS programme.

____

What would have enhanced your ASRS experience?

____

Thank you for your time, your feedback will be invaluable for the development of the ASRS programme.
Please click **'Submit'.**
